# Clinical-Endoscopic Discrepancy in Severe Tonsillar Hypertrophy: When Oropharyngeal Examination Underestimates Airway Obstruction

**DOI:** 10.7759/cureus.108029

**Published:** 2026-04-30

**Authors:** Raysa Mahomed Aboobakar, Gabriel Alexis Magalhães Ibañez, Luiz Guilherme Itimura Mestre

**Affiliations:** 1 Otolaryngology, Universidade do Sul de Santa Catarina, Florianópolis, BRA; 2 Family and Community Medicine, Universidade do Sul de Santa Catarina, Florianópolis, BRA

**Keywords:** airway obstruction, obstructive sleep apnea (osa), palatine tonsil, tonsillar hypertrophy, videolaryngoscopy, voice disorders

## Abstract

Palatine tonsillar hypertrophy is a common cause of upper airway obstruction and is often assessed through oropharyngeal examination using the Brodsky classification. However, this bidimensional method may underestimate the actual tonsillar volume, particularly in cases of atypical anatomy. We report the case of a patient with long-standing rhinophonia associated with dysphonia and vocal fatigue. Oroscopic examination suggested tonsillar hypertrophy. Due to the discrepancy between symptoms and physical examination findings, videolaryngoscopy was performed, revealing more significant airway compromise than initially appreciated. This case highlights the importance of clinical-endoscopic correlation and suggests that discordance between symptoms and oroscopic findings should prompt endoscopic evaluation to avoid the underdiagnosis of clinically relevant airway obstruction.

## Introduction

Palatine tonsillar hypertrophy is a common cause of upper airway obstruction and may be associated with clinical manifestations such as obstructive sleep apnea (OSA), dysphonia, and vocal fatigue, with significant functional and psychosocial impact [1,2]. In clinical practice, initial evaluation is typically performed through direct oropharyngeal examination, often using the Brodsky classification, which categorizes tonsillar size based on the proportion of the oropharyngeal airway occupied (ranging from minimal obstruction to near-complete obstruction) [3]. This method is widely used due to its simplicity and practicality during physical examination.

However, important limitations of this approach have been described. As a bidimensional assessment, oropharyngeal examination may underestimate the true volume of tonsillar tissue, particularly in cases of atypical anatomy where tonsillar enlargement occurs predominantly in medial or caudal directions [3]. In these situations, hypertrophy may extend toward the midline or inferiorly into the oropharyngeal space, reducing airway patency without being fully appreciated on frontal inspection. In addition, the Brodsky classification is subject to interobserver variability, influenced by factors such as tongue position, patient cooperation, and examiner experience [3]. As a result, a discrepancy may arise between clinical findings and symptom severity, potentially leading to the underestimation of upper airway obstruction and delay in diagnosis and appropriate management.

Endoscopic evaluation has been increasingly recognized as a valuable complementary method in the assessment of upper airway obstruction. Techniques such as videolaryngoscopy allow detailed and angular visualization of the oropharynx and larynx, enabling the identification of anatomical alterations not evident on routine examination [4,5]. This approach may improve diagnostic accuracy, particularly in cases where clinical findings are inconclusive or disproportionate to symptom severity.

Although tonsillar hypertrophy is more frequently described in the pediatric population, significant hypertrophy in adults is less common and may be underrecognized, particularly when anatomical variations hinder its identification during routine clinical examination [2]. Given the potential association between upper airway obstruction, OSA, and cardiovascular comorbidities, delayed recognition may have important clinical implications [6].

Reports of clinical-endoscopic discrepancy in tonsillar hypertrophy, particularly in adults, remain limited in the literature. Therefore, the present study aims to describe a case of significant palatine tonsillar hypertrophy underestimated on oropharyngeal examination, with diagnosis clarified by videolaryngoscopy, highlighting the importance of integrating clinical evaluation with complementary diagnostic methods in the assessment of upper airway obstruction.

## Case presentation

A 38-year-old woman with class III obesity (body mass index: 42 kg/m²) and resistant systemic arterial hypertension presented with persistent voice-related complaints. She reported long-standing rhinophonia since childhood, associated with recurrent episodes of dysphonia and vocal fatigue, particularly during increased vocal effort. These symptoms significantly impacted her quality of life, including social interactions and communication.

On initial evaluation, oropharyngeal examination revealed palatine tonsillar hypertrophy classified as Brodsky grade II (corresponding to approximately 25-50% obstruction of the oropharyngeal lumen). No other significant abnormalities were identified on routine physical examination.

However, given the persistence and severity of symptoms, which appeared disproportionate to the physical findings, a discrepancy between clinical presentation and oroscopic assessment was suspected.

For further evaluation, videolaryngoscopy was performed using a 70° rigid endoscope. The examination demonstrated marked bilateral tonsillar hypertrophy with medialization toward the midline and inferior displacement. The tonsillar tissue occupied most of the oropharyngeal lumen and obscured key anatomical structures, including the epiglottis and vocal folds, indicating near-complete airway obstruction (Video 1).

**Video 1:** Videolaryngoscopic view of severe palatine tonsillar hypertrophy with airway compromise This video demonstrates marked bilateral palatine tonsillar hypertrophy with medialization toward the midline and inferior displacement. The tonsillar tissue occupies most of the oropharyngeal lumen, resulting in near-complete obstruction. Key anatomical structures, including the epiglottis and vocal folds, are partially or completely obscured. The findings illustrate the discrepancy between relatively mild oropharyngeal examination and significant airway compromise identified on endoscopic evaluation.

The obstruction appeared predominantly static, with no significant dynamic collapse observed during phonation or respiration. The use of "grade IV" in this context reflects a descriptive approximation of severe obstruction rather than the strict application of the Brodsky classification to endoscopic findings.

No additional structural abnormalities were identified in the larynx.

Based on the endoscopic findings and overall clinical context, the patient was referred for specialized otolaryngological evaluation, with indication for tonsillectomy.

## Discussion

This case highlights a clinically relevant diagnostic challenge in the evaluation of patients with suspected upper airway obstruction: the presence of persistent symptoms that are not proportionally reflected in initial physical examination findings. Palatine tonsillar hypertrophy is a common cause of oropharyngeal obstruction and may be associated with respiratory symptoms, voice changes, and sleep disturbances [1]. In clinical practice, initial evaluation is often based on oropharyngeal examination using the Brodsky classification, a widely adopted method for estimating tonsillar size [2].

However, this approach has important limitations. The Brodsky classification is based on a frontal, bidimensional assessment and does not adequately reflect the three-dimensional volume of tonsillar tissue [2]. This limitation becomes particularly relevant in cases where hypertrophy occurs predominantly in medial or caudal directions. In such scenarios, tonsillar tissue may extend toward the midline or inferiorly within the oropharyngeal space, reducing airway patency without appearing markedly enlarged on routine examination.

Additionally, other potential causes of upper airway obstruction must be considered, including obesity-related airway narrowing, lingual tonsil hypertrophy, and laryngeal pathology [1,3]. In the present case, obesity represents a significant confounding factor, as it is strongly associated with upper airway obstruction and sleep-disordered breathing [6,7].

Endoscopic evaluation plays a crucial role in such cases. Videolaryngoscopy allows a detailed and angular visualization of the oropharynx and larynx, enabling the identification of anatomical alterations not evident during routine examination [3,4]. As demonstrated in this case, it can reveal clinically significant airway compromise that may be underestimated on oropharyngeal inspection alone.

Although tonsillar hypertrophy is more commonly described in pediatric populations, significant cases in adults may be underrecognized, particularly when anatomical variations hinder detection during routine clinical examination [5]. In this context, it is important to note that tonsillar size alone does not necessarily correlate with the severity of airway obstruction or OSA [5].

OSA is characterized by recurrent episodes of partial or complete upper airway obstruction during sleep, leading to intermittent hypoxia and sympathetic activation [5]. These mechanisms may contribute to cardiovascular consequences, including resistant hypertension [6]. However, in the present case, OSA was not formally evaluated, and no objective data, such as apnea-hypopnea index, are available. Therefore, these associations should be interpreted with caution and not directly inferred from the findings presented.

The identification of potentially treatable anatomical causes of upper airway obstruction remains clinically relevant, particularly in patients with comorbid conditions such as obesity and resistant hypertension. In such cases, early recognition may contribute to symptom improvement and guide appropriate management.

Clinical features such as persistent symptoms disproportionate to physical findings, chronic dysphonia, and risk factors, including obesity, should raise suspicion for further investigation. In these scenarios, complementary endoscopic evaluation plays a key role in identifying anatomical alterations not evident on routine examination.

This case highlights that bidimensional assessment of the oropharynx may underestimate airway obstruction in patients with atypical anatomy. When clinical findings do not correspond to symptom severity, further evaluation using endoscopic methods should be considered to improve diagnostic accuracy and guide appropriate management.

## Conclusions

Oropharyngeal examination remains a fundamental tool in the initial assessment of tonsillar hypertrophy; however, its limitations should be recognized, particularly when physical findings do not adequately explain symptom severity. This case highlights the importance of recognizing clinical-endoscopic discrepancy in patients with atypical tonsillar anatomy, in whom significant airway obstruction may be underestimated on routine examination.

In such scenarios, complementary endoscopic evaluation may provide a more accurate assessment of airway compromise and support appropriate clinical decision-making, particularly in patients with persistent or disproportionate symptoms and relevant risk factors.
